# Isolation and structural characterization of bioactive compound from *Aristolochia sprucei* aqueous extract with anti-myotoxic activity

**DOI:** 10.1016/j.toxcx.2020.100049

**Published:** 2020-06-20

**Authors:** Isela I. González Rodríguez, Aleff F. Francisco, Leandro S. Moreira-Dill, Aristides Quintero, César L.S. Guimarães, Carlos A.H. Fernandes, Agnes A.S. Takeda, Fernando B. Zanchi, Cléopatra A.S. Caldeira, Paulo S. Pereira, Marcos R.M. Fontes, Juliana P. Zuliani, Andreimar M. Soares

**Affiliations:** a Faculdade de Ciências Farmacêuticas de Ribeirão Preto, FCFRP, Universidade de São Paulo, USP, Ribeirão Preto, SP, Brazil; b Laboratório de Biotecnologia de Proteínas e Compostos Bioativos da Amazônia Ocidental, LaBioProt, Centro de Estudos de Biomoléculas Aplicadas à Saúde, CEBio, Fundação Oswaldo Cruz, FIOCRUZ, Unidade Rondônia e Universidade Federal de Rondônia, UNIR, Porto Velho, RO, Brazil; c Centro de Informaciones e Investigaciones Toxicológicas y Químicas Aplicadas (CEIITOXQUIA) and Departamento de Química, FCNYE, Universidad Autónoma de Chiriquí, UNACHI, David, Panama; d Instituto Brasileiro Do Meio Ambiente e Dos Recursos Naturais Renováveis, IBAMA, Porto Velho, RO, Brazil; e Unidade de Biotecnologia, Universidade de Ribeirão Preto, UNAERP, Ribeirão Preto, SP, Brazil; f Instituto Federal de Goiás, IFG, Goiania, GO, Brazil; g Departamento de Física e Biofísica, Instituto de Biociências, Universidade Estadual Paulista, UNESP, Botucatu, SP, Brazil; h Laboratório de Imunologia Celular Aplicada a Saúde, Fundação Oswaldo Cruz, FIOCRUZ, Unidade Rondônia, Porto Velho, RO, Brazil; i Centro Universitário São Lucas, UniSL, Porto Velho, RO, Brazil; j Instituto Nacional de Ciência e Tecnologia Em Epidemiologia da Amazônia Ocidental, INCT – EpiAmO, Brazil

**Keywords:** *Aristolochia sprucei*, Aristolochic acid, Antivenom medicinal plants, Myotoxin inhibitor, Snake venoms, Phospholipases A_2_

## Abstract

A bioactive compound isolated from the stem extract of *Aristolochia sprucei* through High Performance Liquid Chromatography (HPLC) was identified via Nuclear Magnetic Resonance (NMR) as the aristolochic acid (AA). This compound showed an inhibitory effect over the myotoxic activity of *Bothrops jararacussu* and *Bothrops asper* venoms, being also effective against the indirect hemolytic activity of *B. asper* venom. Besides, AA also inhibited the myotoxic activity of BthTX-I and MTX-II with an efficiency greater than 60% against both myotoxins. Docking predictions revealed an interesting mechanism, through which the AA displays an interaction profile consistent with its inhibiting abilities, binding to both active and putative sites of svPLA_2_. Overall, the present findings indicate that AA may bind to critical regions of myotoxic Asp 49 and Lys49-PLA_2_s from snake venoms, highlighting the relevance of domains comprising the active and putative sites to inhibit these toxins.

## Highlights

• Aristolochic acid inhibits myotoxicity of *Bothrops jararacussu* and *B. asper* venoms.
• Aristolochic acid partially inhibits the phospholipase A_2_ activity of *B. asper* venom.
• Aristolochic acid also inhibits myotoxicity of myotoxins.
• Docking predictions revealed that Aristolochic acid binds to myotoxins putative sites.

## 1 Introduction

Snake bite envenomation is a global public health problem, especially in tropical and subtropical areas, which makes the search for antivenom agents and the knowledge of their toxic-pharmacological effects of great relevance (Gutiérrez et al., 2006, Kasturiratne et al., 2008, Xiao et al., 2017).

Most of snake venom constituents are proteins, corresponding to 90–95% of the dry venom, where the presence of enzymatic activity is often observed (Angulo and Lomonte, 2009, Koh et al., 2006a, Koh et al., 2006b). The literature describes several families of toxins identified in snake venoms, such as: Phospholipases A_2_, Three-finger toxins, Metalloproteases, Serine proteases, Kunitz peptides, L-amino acid oxidases, Cysteine-rich secretory proteins, C-type lectins/snaclecs, Disintegrins and Natriuretic peptides (Tasoulis and Isbister, 2017, Warrell, 2010). Amidst all enzymes present in snake venoms, the Phospholipases A_2_ (PLA_2_s) are among the most widespread and studied, due to its physical-chemical properties, biological importance, and biotechnological potential (Aloulou et al., 2018, Kini, 2003, Samy et al., 2014). In fact, Phospholipases A_2_ from snake venom (svPLA_2_s) have a great variety of pharmacologic and/or toxic effects, such as: myonecrotic, anticoagulation, platelet aggregation inhibition, neurotoxic, cardiotoxic, hypotensor and edema (Burke and Dennis, 2009, Gutiérrez and Lomonte, 2013, Harris and Scott-Davey, 2013, Péterfi et al., 2019, Xiong and Huang, 2018).

Many plants have been described all over the world as been used by traditional medicine to treat snake bites. The continuous search and identification of new compounds that can be useful as alternatives and/or complementation to serum therapy, stands as a challenge of great importance (Félix-Silva et al., 2017, Gómez-Betancur et al., 2019, Hage-Melim et al., 2013, Jorge et al., 2019, Marcussi et al., 2007, Santhosh et al., 2013, Soares et al., 2009, Soares et al., 2005, Upasani et al., 2018).

Studies with extracts and secondary metabolites from *Aristolochia* species, comprising mostly nitrogen compounds such as Aristolochic Acid (AA) and aristolochin, showed important biological properties, for instance: immunostimulatory, anti-inflammatory and PLA_2_s inhibition (Chung et al., 2011, Coe and Anderson, 2005; Carlos A. H. Fernandes et al., 2015a, Fernandes et al., 2015b, Giovannini and Howes, 2017). It's worth mentioning, that there are also challenging aspects involved in the implementation of AA as a therapeutic agent, such as the extensively reported nephrotoxic and carcinogenic side effects of AA. Nevertheless, the protective effects offered by AA against snake venoms are promising, especially when considering the available strategies to solve AA complications, such as the concomitant administration of compounds to treat AA toxic outcomes or structural modifications aiming the design of safer AA derivatives with optimized affinity over its molecular targets (Anger et al., 2020, Zhou et al., 2019). Thus, the present work has evaluated the effects of AA isolated from *Aristolochia sprucei*, against bothropic venoms and isolated toxins, paving the way for further exploration of AA antivenom potential as an alternative for increasing the efficacy of future snakebite therapies.

## 2 Material and methods

### 2.1 Venom and myotoxins

Venom was obtained from *Bothrops asper* adult snakes from Panama, captured in Caldera (latitude: 8°39′6.37″N; longitude: 82°23′30.18″ O) and Goméz (San Andrés) (latitude 8°35′12.04″N, longitude 82°44′16.38″O) in the province of Chiriquí, and in Gamboa (latitude 9°07‘17.81″N, longitude 79°41′36.94″O) in the province of Panamá. The collecting spot was marked using a global positioning system (GPS) model Garmin 60CSx.

The specimens were kept in captivity in the Gamboa Rain Forest Resort serpentarium, in Panamá and validated with the collaboration of Dr. Wilson Fernandes (Herpetology Laboratory Chief, Butantan Institute, São Paulo, Brazil) and of Prof. Dr. Ida Sano-Martins (Physiopathology Laboratory Chief of the Butantan Institute – SP). The crude venom was obtained through manual manipulation of the snakes on a collecting cup covered with parafilm. The venom was centrifuged at 1.000×*g* for 15 min and the supernatant lyophilized and stored at −20 °C with the collaboration of Prof. Nora Ortiz de Moreno (Microbiology department, Medicine College, University of Panamá).

*Bothrops jararacussu* venom was acquired from Serpentário de Proteínas Bioativas Ltda, Batatais-SP and kept refrigerated (8 °C) in the Bank of Amazon Venoms at the Center of Biomolecular Studies Applied to Health, CEBIO-UNIR-FIOCRUZ-RO (authorization: CGEN/AFCAB61 and SISBIO 64385–1).

### 2.2 Animals

Male Swiss mice weighting about 18–22 g were used for the venom biological activity study. The animals were acquired from the Central Vivarium of the Ribeirão Preto campus of University of São Paulo (USP) and kept at adequate places and conditions according to the Brazilian College of Animal Experimentation (COBEA) rules and with the approval of the Ethics Committee in the Use of Animals of the São Paulo University (CEUA No. January 08, 1096.53.1).

### 2.3 Vegetal material

The plant collections were conducted with the Environment National Authority of Panamá (ANAM) approval (Scientific permission of collection No. SE/P-50-07) (Attachment No. 2) at Serra Llorona (latitude: 9°17’12.49″N; longitude: 79°37′02.81″O) and Santa Rita (latitude: 9°20′34.90″ N; longitude: 79°48′21.27″) in the Colón Province. Biologist Alex Martinez (Pharmacognosy of Panama Flora FLORPAN, University of Panamá) identified the voucher herbarium specimens. One voucher of each species (Register8234a) was deposited in the Biology School Herbarium of the Natural and Exact Science College, University of Panamá, under the direction of Professor Mireya Correa.

After the collection, the *in natura* material was separated into leaves and stems. In order to avoid degradation of the chemical compounds, the material was dried for a week by ventilation, providing air to the samples. The samples, which did not dry completely, were taken to the Pharmacognosy of the Panamanian Flora Research Center (CIFLORPAN) of the Pharmacy College of the University of Panama. The plants stayed for 24 h in a dry oven Equaterm at 50 °C. Subsequently, the material was grinded in a Thomas-Wiley (Laboratory Mill Model 4) knife grinder for pulverization of the material with thick cut and 50 mesh sieving to adequate storage.

The pulverized material was exported to the Brazil Toxicological Biochemistry Laboratory of the Pharmaceutical Sciences College of Ribeirão Preto with the approval of the Panama's Environment National Authority (ANAM) (Export Permission No. SEX/P-66-07). The packed plant material, following the above reported procedures, was submitted to hot water extraction for 24 h and kept from light. The aqueous extracts were filtered and lyophilized. The filtrated residues were dried up in an oven and submitted to another extraction with different polarity solvents (methanol and ethyl acetate), with subsequent chromatographic extraction to obtain purified fractions.

### 2.4 Aristolochic acid isolation

The *Aristolochia sprucei* crude extract, from leaves and stems with ethyl acetate was analyzed by HPLC using a Supelco™ LC18 (25 cm × 4.6 mm) analytical column in a Shimadzu chromatograph with diode array detector (model CLASS-LC10). As standard, the commercial AA I (C_17_H_11_NO_7_, PM. 341.27 g/mol) from Sigma Aldrich (No. A5512) was used. The material was analyzed through polar and non-polar conditions, using a linear gradient as follows: **Polar Substances**: the run time was set in 40 min using 1 mL/min flow, fractions were collected in 3 mL/tube, monitored in λ = 254 nm, with an increasing gradient of 10–66% MeOH in the first 32 min, followed by 66–10% MeOH during 32–35 min in a decreasing gradient, and an isocratic flow of 10% MeOH in the time left. **Apolar substances**: the run time lasted 35 min with a flow rate of 1 mL/min, fractions were collected in 3 mL/tube, monitored in the wave-lengths of 210 and 330 nm, using an increasing gradient in the first 15 min of 40–80% MeOH, with a change to 80–100% MeOH in the 15–20 min period, followed by 100% MeOH in the 20–30 min and a decreasing of 100–40% MeOH 30–33 min, ending the run with an isocratic flow of 40% MeOH.

### 2.5 Aristolochic acid NMR identification

The fractions separated by HPLC were analyzed by NMR, using 1–5 mg of the polar samples dissolved in 500 μL of deuteride chloroform and deuteride dimethylsulphoxide inside resonance tubes. The Nuclear Magnetic Resonance spectra ^1^H and ^13^C were obtained from a Bruker Avance DRX-500 [500.134 MHz (^1^H) e 125.772 MHz (^13^C)] spectrometer, using deuteride chloroform (CDCl_3_) or deuteride dimethylsulphoxide (DMSO‑*d*_6_) as solvent and tetramethysilane (TMS) as internal reference standard. The chemical shifts (δ) were obtained in parts per million (ppm) and the coupling constants (*J*) in Hertz. The spectral data represent the average of 50 sweeps (0.2 s/sweep).

### 2.6 Analysis of the interaction between inhibitor and myotoxin by circular dichroism

Circular dichroism (CD) measurements were carried out in a JASCO model J-815 (JASCO Inc., Tokyo, Japan) CD spectropolarimeter equipped with a peltier thermo-controller within a spectral range of 185–260 nm. The experiments were performed at 293 K using an optical path-length of 0.5 mm with a 100 nm/min scanning speed, a bandwidth of 2 nm, response time of 1 s and a data pitch of 0.5 nm. Thirty-five spectra were acquired, averaged and the resultant spectra were normalized to residual molar ellipticity [θ]. Lyophilized samples of BthTX-II and MjTX-I were dissolved at ultrapure deionized water at concentration of 250 μg/mL that were verified in a UV–vis spectrophotometer NanoDrop 2000c (Thermo Scientific, Waltham, USA) using molar extinction coefficients from both proteins. For the measurements with AA, a ratio of 8 AA molecules to 1 molecule of protein was used. The x and y coordinates of all spectra presented were plotted in a graph drawn with the *Origin* 8.0 software.

### 2.7 Molecular docking

The myotoxins BthTX-I and BthTX-II from *B. jararacussu*, Myotoxin II from *B. asper* and MjTX-I from *B. moojeni* coordinates were obtained in the Protein Data Bank (respectively PDB ID codes: 3CXI, 3JR8, 1Y4L, and 3T0R) and were submitted to an energy minimization on a partial implementation of the GROMOS 96 force field, with all computations done in vacuum, without reaction field (Schuler et al., 2001). The AA structure used was extracted from PubChem/NCBI (CID:2236) and minimized using a conjugate gradient algorithm with Universal Force Field (UFF) applying 1000 steps of minimization through PyRx (Dallakyan and Olson, 2015). The minimized inhibitor was docked into the PLA_2_s using AutoDock 4.2 (Morris et al., 2009). After the energy minimization and targets preparation, the grid maps were generated using AutoGrid, centralized on the histidine 48 α carbon of the chain A in each target, with a dimension proportional enough to involve the macromolecules active and putative sites (3CXI: 150 × 150 × 150; 3JR8: 71 × 71 × 71; 1YL4: 180 × 180 × 180; 3T0R: 130 × 130 × 130) and a spacing of 0.375 Å between grid points. In the next subsequent step, AutoDock files were prepared, and docking simulations were carried out using Lamarckian Genetic Algorithm (LGA), with an initial population of 150 randomly placed individuals, 250 runs of Genetic algorithm, a maximum number of 2.5 × 10^6^ energy evaluations, a mutation rate of 0.02, a crossover of 0.08 and an elitism value of 1. The analysis and figures were made on the UCSF Chimera (Pettersen et al., 2004).

### 2.8 Neutralization protocol

Neutralization assays were done using a venom/extract ratio of 1:2, 1:5, 1:10, 1:20 e 1:30 (w/w), with a pre-incubation time of 30 min at 37 °C. After this period, the enzymatic, toxic and pharmacologic activities of the crude venom and the myotoxins were done as described below, keeping the following groups: (a): crude venom + PBS (positive control); (b): crude venom + vegetal extract; (c): vegetal extract + PBS (negative control); (d): DMSO + PBS (diluent control).

### 2.9 Pharmacologic, toxic and enzymatic activities

#### 2.9.1 Phospholipase activity

The phospholipase activity was tested by the indirect radial hemolysis method, performed on plates (Gutiérrez et al., 1988). The samples consisted of crude venom or myotoxin, previously incubated with the different extracts and fractions for 30 min at 37 °C, in different proportions. The gels containing the samples were placed in an oven at 37 °C for 12 h. The translucent halo formations, around the gel orifice, indicate the activity and were measured (cm) to quantify the phospholipase activity. Determinations were done in quadruplets. Indirect phospholipase activity was defined as the diameter in millimeters of the hemolysis area (mm ± SD).

#### 2.9.2 Myotoxic activity

Myotoxicity was induced by i. m. (intramuscular) injection at the gastrocnemius right muscle of *Swiss* male mice (18–22 g), with 50 μL of different concentrations of the crude venom or myotoxin (25 μg), diluted in PBS and previously incubated with different extracts and fractions, during 30 min at 37 °C, in different proportions. Three hours later, the blood was collected, by incision at the extremity of the tail, in heparinized capillaries and immediately centrifuged at 480×*g* for 20 min. The activity of the creatine kinase enzyme was determined using 4 μL of plasma incubated with 1 mL of the CK-UV kinetic kit reactive (Bioclin) dissolved in distilled water for 3 min at 37 °C, with spectrophotometer readings at 340 nm. The activity was expressed in units/liter (mean ± SD). One unit consists of the phosphorylation result in one nanomol (nmol) of creatine per minute (A M. Soares et al., 2000a, Soares et al., 2000b; A. M. Soares et al., 2000a, Soares et al., 2000b).

### 2.10 Statistical analysis

Resulting data were statistically analyzed, with the results expression being done by the mean ± standard deviation (SD) and the significance levels considered in the reliance interval p < 0.05.

## 3 Results

**Fig. 1:**
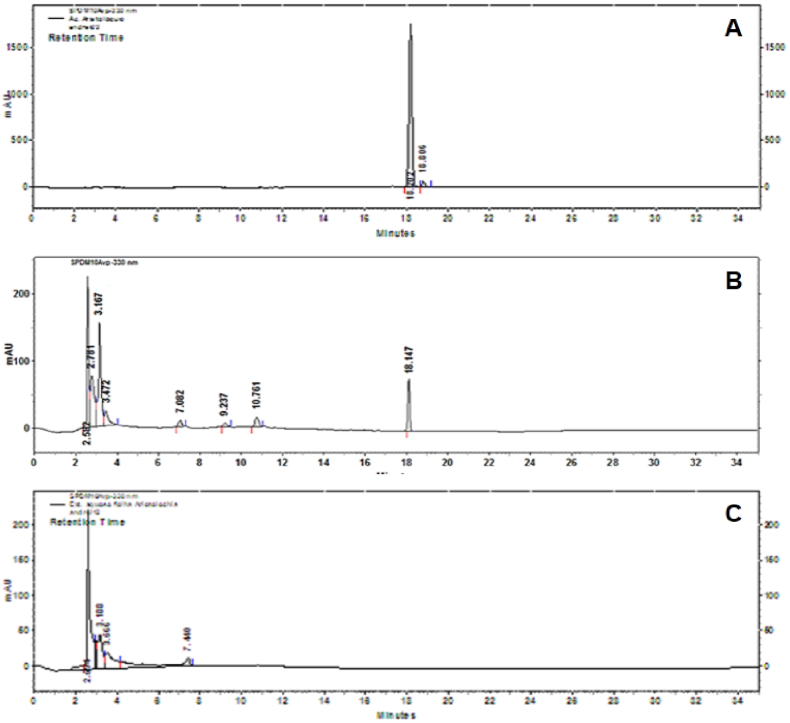
*Aristolochia sprucei* (stem and leaf) crude extract in ethyl acetate analysis by HPLC using a Supelco™ LC18 (25 cm × 4.6 mm, Supelco®) analytic column in a Shimadzu Chromatograph with diode array detector (model CLASS-LC10). (A) AA I chromatogram control, Sigma commercial standard. (B) *A. sprucei* stem extract chromatogram. The Arrow indicates the presence of compatible signal with AA I. (C) *A. spruce* leaf extract chromatogram.

Fig. 1 shows the AA chromatograms, the standard (Fig. 1A), stem's extracts (Fig. 1B) and leaf (Fig. 1C) of *Aristolochia sprucei* in ethyl acetate. The analysis conditions provided signals with good resolution and symmetry, maintaining a reasonable time analysis. The chromatographic profile correspondent to the commercial standard demonstrated a retention time of 18.20 min and an absorption peak at 330 nm (Fig. 1A). The stem extract chromatogram (Fig. 1B) showed several peaks, with a peak specifically at 18.14 min presenting an absorption profile similar to the one observed for the AA standard. On the other hand, the leaves extract chromatogram (Fig. 1C) showed no signal response in the 18 min range, even though, there were peaks at 3.16, 3.66 and 7.44 min.

**Fig. 2:**
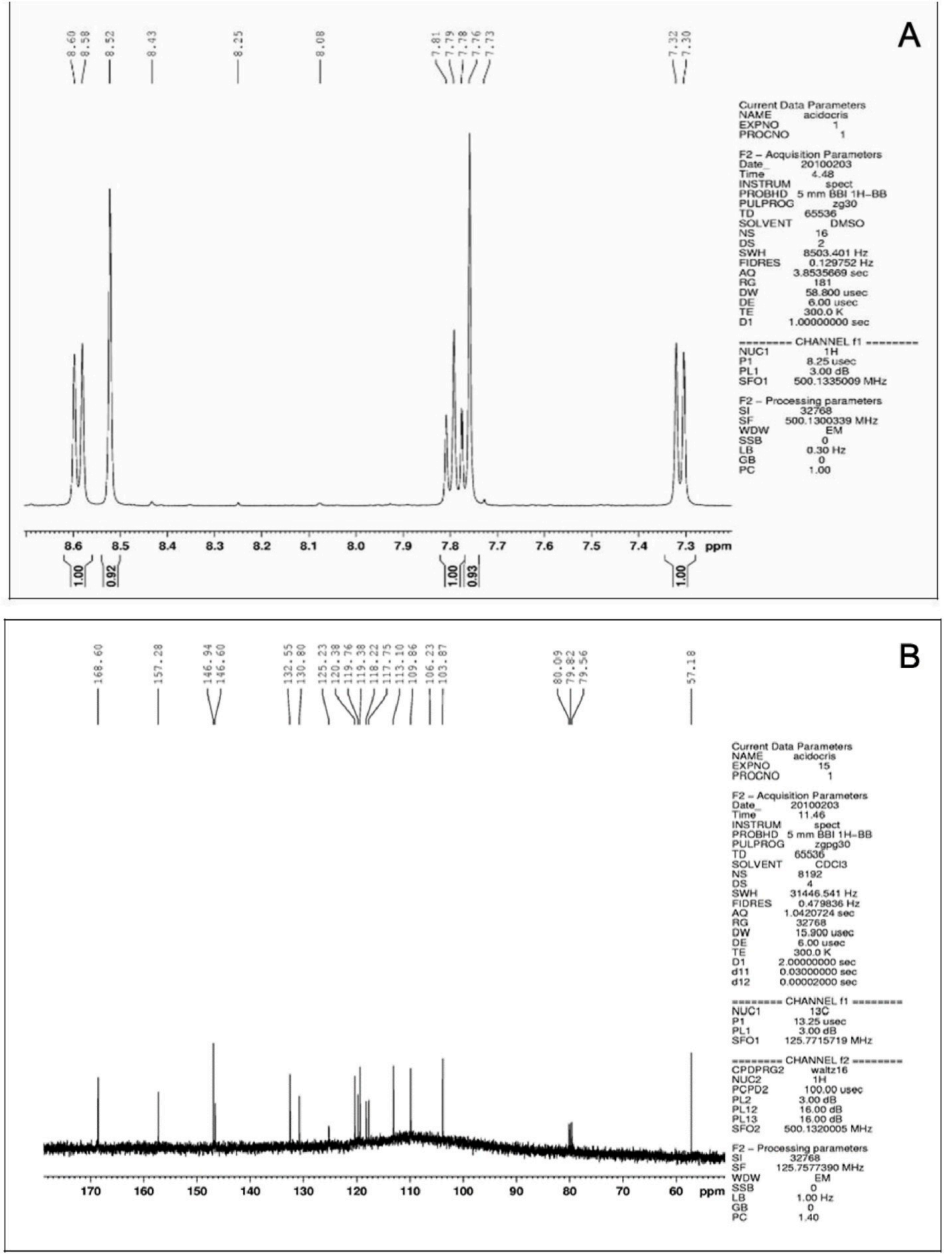
NMR proton (NMR ^1^H) and carbon (NMR ^13^C) spectroscopic analysis of the compound isolated by HPLC. (A) ^1^H NMR proton spectra (DMSO, 500 MHz, δ). (B) ^13^C NMR (CDCl_3_, 126 MHz, δ) carbon spectra.

**Fig. 3:**
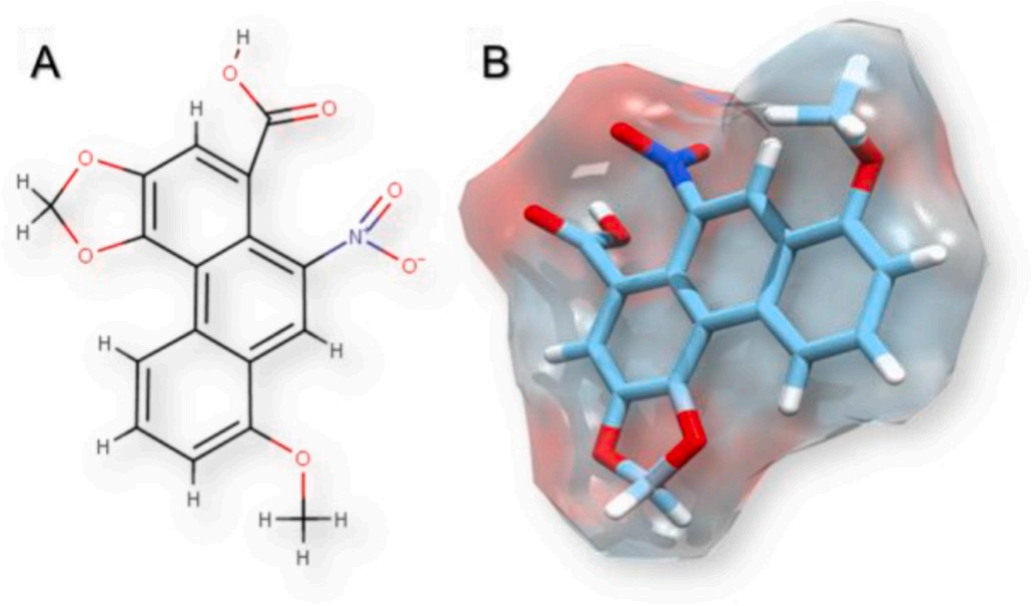
Chemical structure of AA I. (A) Bidimensional chemical structure. (B) Tridimensional molecular structure in a protonated state, drawn in UCSF Chimera 1.11.2.

Fig. 2 (A and B) shows the proton (NMR ^1^H) and carbon (NMR ^13^C) in the NMR spectroscopy analysis of the compound isolated by HPLC (Fig. 1B). The analyzed spectra showed 17 signals for carbon atoms, 11 signals for hydrogen atoms and two substituents: OCH_2_O and OCH_3_, which corresponds structurally to the AA (Fig. 3). The chemical shifts (δ) of the compound isolated by HPLC (Fig. 1B) were compared with the chemical shifts patterns informed by (Nascimento and Lopes, 2003) for aristolochic acid (Table 1).

**Table 1:** Comparison of the chemical shifts obtained by NMR in the sample with literature standards of the AAI.

Carbon	Sample	4^a^^,^^b^	Sample	4^a^^,^^c^
1	**125.23**	**124.5**		
2	**113.10**	**112.2**	**7.76 s**	**7.80 s**
3	**146.60**	**146.0**		
4	146.94*	**146.2**		
4^a^	**117.75**	**116.9**		
4 b	**130.80**	**129.8**		
5	**119.38**	**118.4**	**8.59 d**	**8.66 d**
6	**132.55**	**131.5**	**7.79 t**	**7.68 t**
7	**109.86**	**108.7**	**7.31 d**	**7.07 d**
8	**157.28**	**156.3**		
8^a^	**119.76**	**118.8**		
9	**120.38**	**119.5**	**8.52 s**	**8.81 s**
10	146.94*	**145.7**		
10^a^	**118.22**	**117.3**		
11	**168.60**	**167.9**		
OCH_2_O	**103.87**	**103.0**	**6.43**	**6.34 s**
OCH_3_	**57.18**	**56.2**	**4.00**	**4.01 s**

Obs.: There was a shift of the signals of ^13^C NMR in comparison with literature data.

a Data from literature (Nascimento and Lopes, 2003).

b Solvent: DMSO‑*d*_6._

c Solvent: CDCl_3_.

**Fig. 4:**
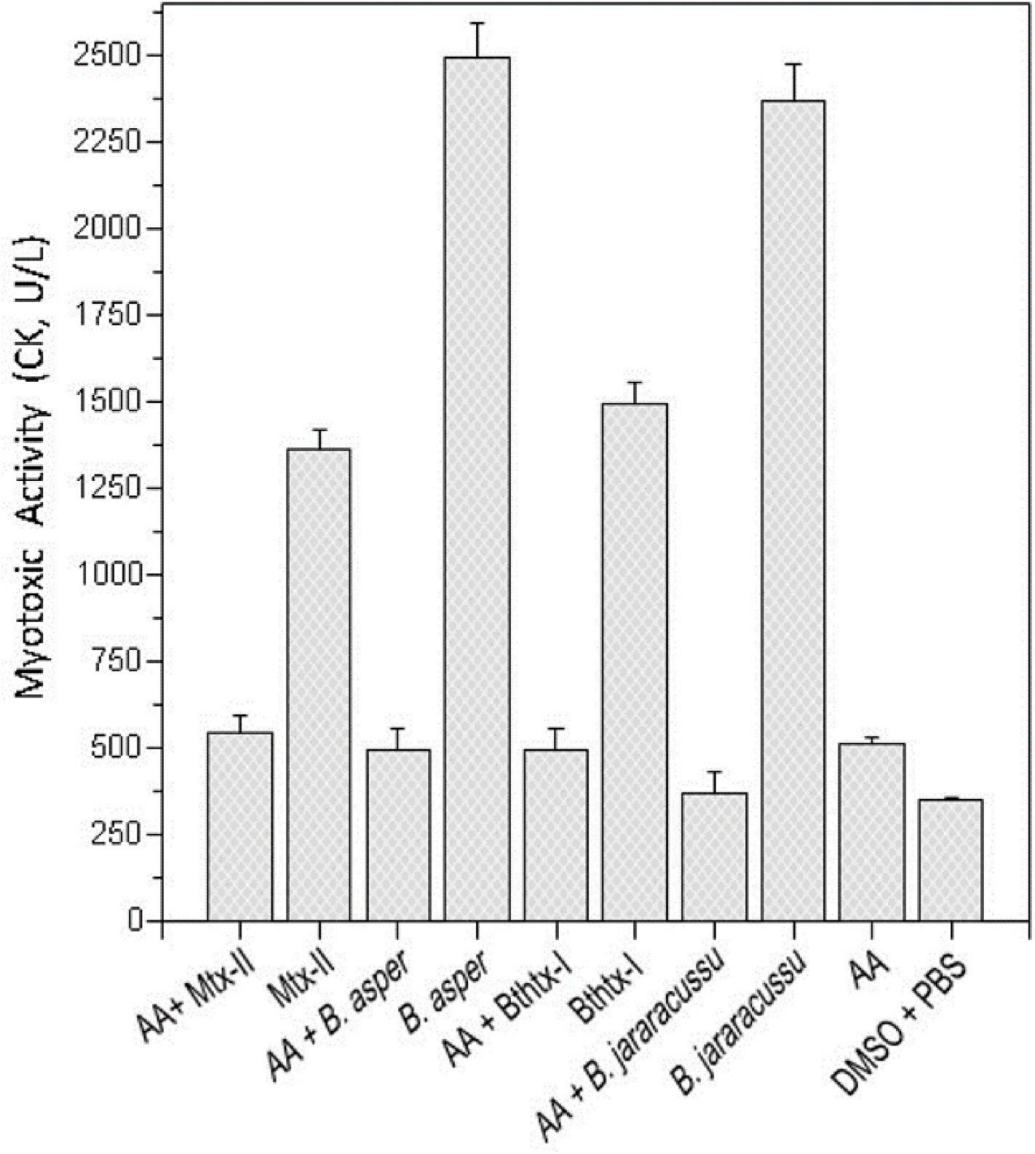
Venoms (*B. jararacussu*; *B. asper*) and myotoxins (BthTX-II; MTX-II) myotoxic activity inhibition by AA in 1:30 ratio (w/w) previously incubated for 30 min at 37 °C. Results expressed by the mean ± S.D. (n = 5).

**Fig. 5:**
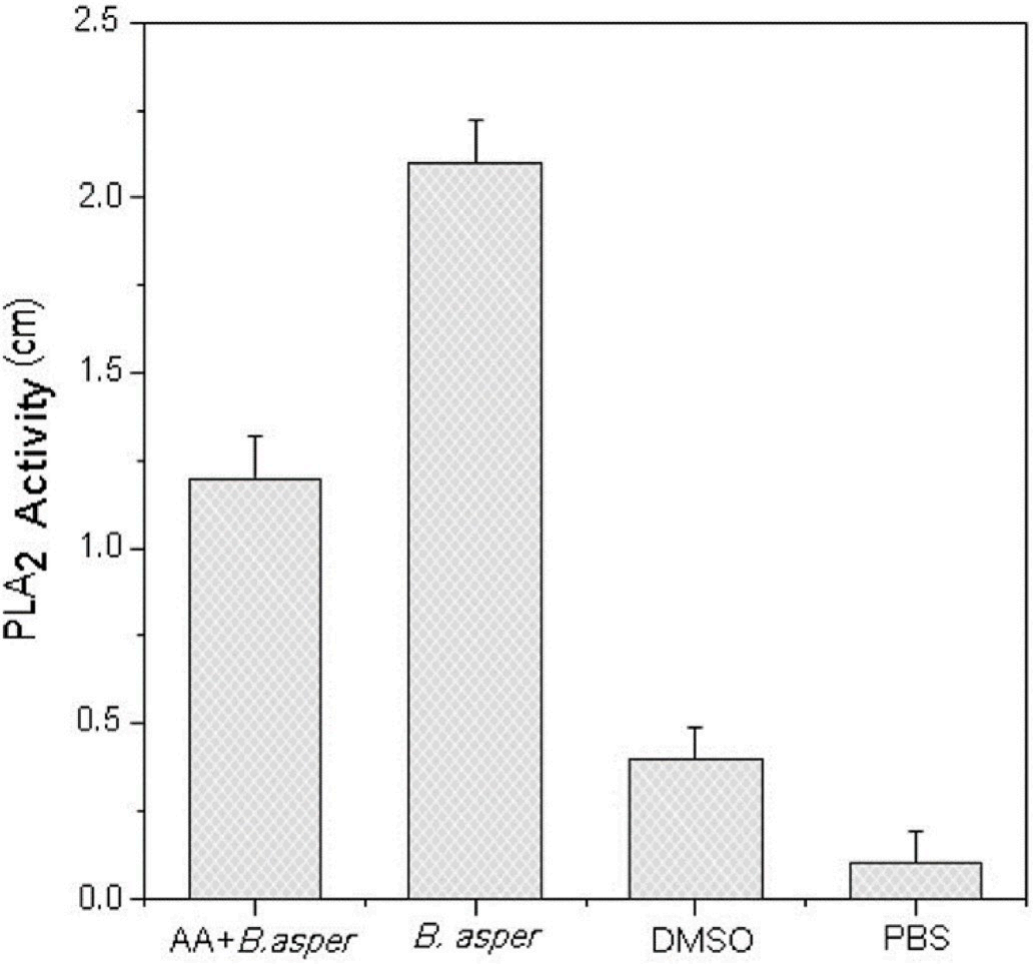
*B. asper* venom phospholipase activity inhibition by the AA isolated from *A. sprucei* in 1:30 ratio (w/w). Results expressed by the mean ± S.D. (n = 3).

*B. asper* and *B. jararacussu* crude venoms and the respective isolated toxins, Myotoxin-II and BthTX-I myotoxic activity were inhibited by AA (Fig. 4). The AA exhibited an inhibition of 85% over the myotoxic activity of *B. jararacussu* venom, followed by 80% for *B. asper* venom, 64% for BthTX-I and 60% for Myotoxin-II. Fig. 5 shows the inhibition effect of AA over PLA_2_ activity induced by *B. asper* venom.

**Fig. 6:**
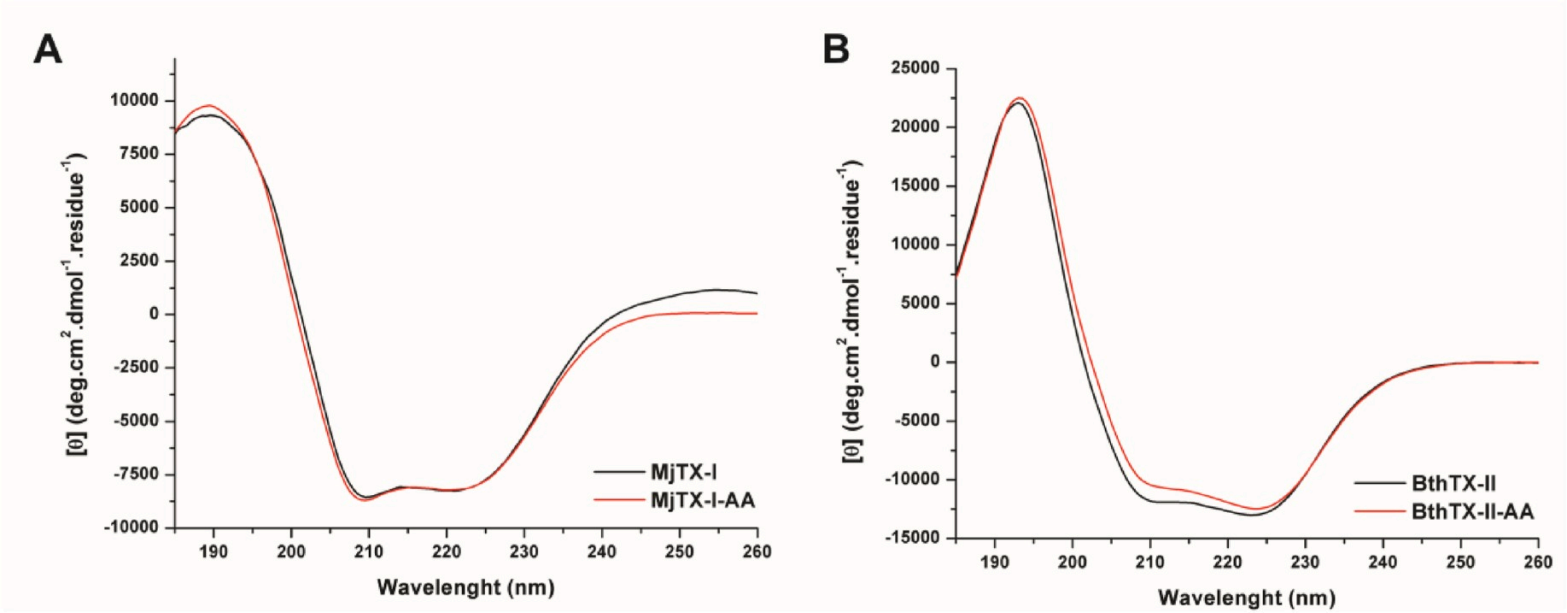
Circular dichroism spectroscopy of the interaction between the AA and the isolated myotoxins. Spectra of the MjTX-I from *B. moojeni* (A) and BthTX-II from *B. jararacussu* (B) in the absence (black lines) and in presence (red lines) of AA from *A. sprucei*.

The circular dichroism spectra of MjTX-I from *B. moojeni* and BthTX-II from *B. jararacussu* are shown in Fig. 6 (A and B), in the presence and absence of AA from *A. sprucei*. AA does not cause any significant secondary structural changes on MjTX-I or BthTX-II, as it can be observed in the CD spectrum in absence of AA, which exhibits a high similarity to the spectrum in its presence (Fig. 6A).

**Fig. 7:**
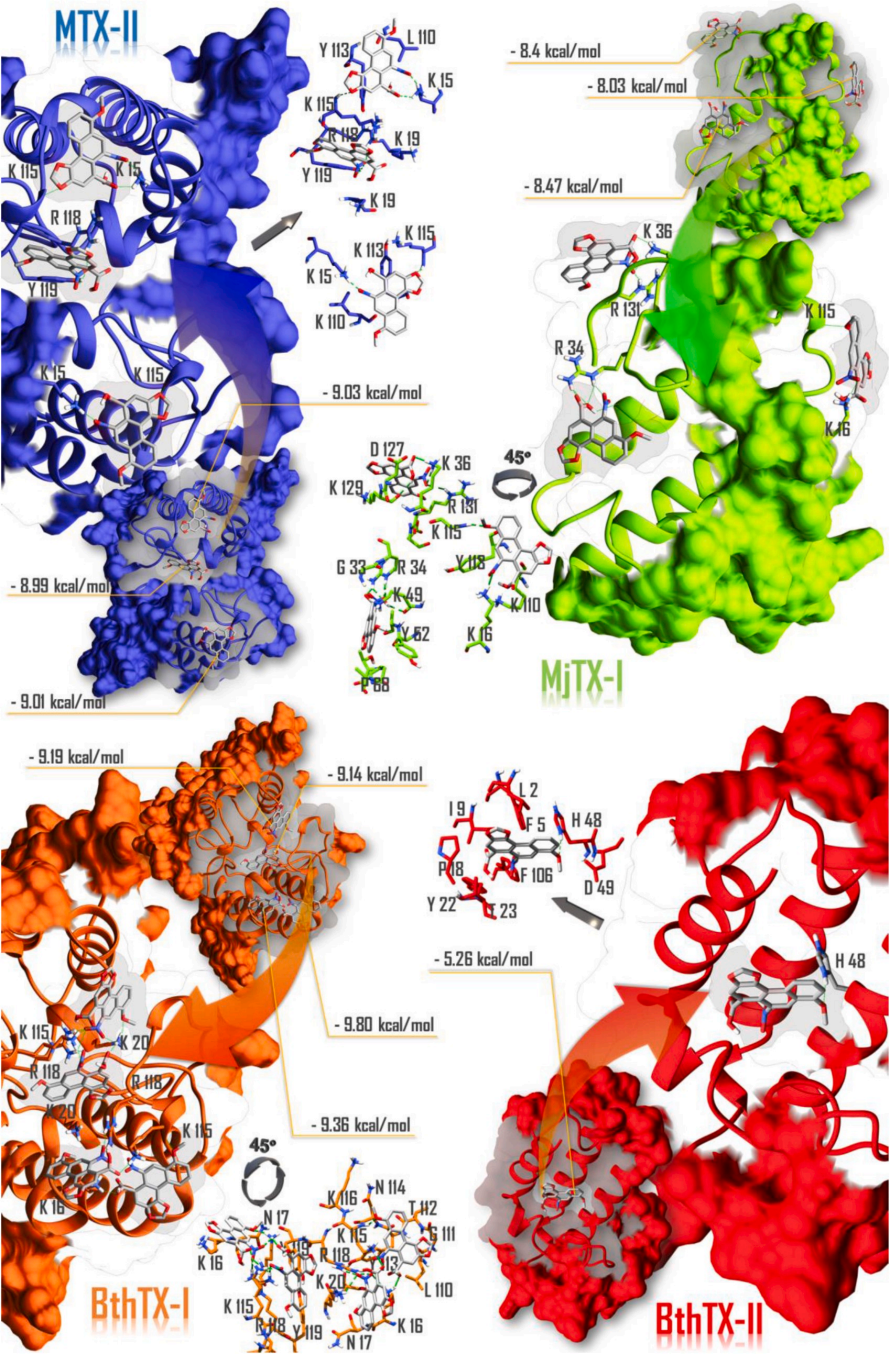
Complexes predicted through docking for the interactions between AA and svPLA_2_s (dimeric MTX-II from *B. asper*; monomeric MjTX-I from *B. moojeni*; BthTX-I dimer from *B. jararacussu*; BthTX-II monomer from *B. jararacussu*). The complexes are shown together with its enlarged AA biding sites, highlighting only side chains making hydrogen bonds (green dashed lines) with AAG. A contact map (residues in sticks matching the color of its corresponding svPLA_2_, and grey sticks for AAG) displaying the nearest amino acids interfacing with AA was included side by side with each of its respective complex of origin. All binding energies (kcal/mol) are pointed out on yellow lines linked to each respective AA docking pose.

The *in silico* molecular interactions between the svPLA_2_s and AA were assessed through hydrogen bonds formation, electrostatic and hydrophobic interactions between the ligand and targets residues. The AA docking results against each myotoxin showed over 30 poses with favorable energy coupling in the catalytic site, MDoS (membrane docking site) and the C-terminal portion. The more predominant poses with lower ΔG are shown (Fig. 7).

## 4 Discussion

In this work, portions of *A. sprucei* extracts of showing greater neutralizing ability over the toxic effects of bothropic venom were selected for further analysis. The isolation of active compounds from *A. sprucei* extracts was performed using chromatographic techniques chosen considering the reference substances described in other works and their purifications protocols. The 23 fractions isolated by the combination of column and thin layer chromatography techniques, inhibited in different measures, the indirect hemolytic activity of the *B. asper* venom (results not shown). These results indicated that *A. sprucei* had phospholipase inhibitory potential, with a likelihood that the isolated compounds could be flavonoids with the capability to inhibit the phospholipase activity, or terpenes, such as the AA, that has a proven antivenom activity (Nascimento and Lopes, 2003).

There are few studies identifying, isolating and characterizing vegetal extracts active compounds against *B. asper* venom. Among which, are the *Piper umbellatum* and *P. peltatum* extracts, that completely inhibited the activity of myotoxin I (MTX-I) isolated from the *B. asper* venom. Fractionation to obtain the active principle, showed a compound identified as 4-nerolidylcatecol. The isolated compound inhibited the PLA_2_ activity of MTX-I, with an average inhibitory concentration (IC_50_) of 987 mM, and has significantly reduced the myotoxicity in mice. When administered *in situ*, immediately after injecting the MTX-I, its inhibitory capability was substantially lower or insignificant (Mors et al., 2000).

*Draconitium dubium* showed protective effect over the lethal, inflammatory, coagulant and hemolytic activities of *B. asper* venom. When treated with 500 and 1000 μg/g of *D. dubium* extract, the mice survived to lethal doses of venom (Núñez et al., 2005). The aqueous extract of *Pentaclethra macroloba* has demonstrated inhibitory properties against hemorrhagic and edematogenic activities of many *Bothrops* spp. Genus snake venoms, including *B. atrox* and *B. asper* (Caro et al., 2017).

*A. sprucei* stem extract chromatographic analysis in ethyl acetate showed a signal with symmetry, resolution and reasonable time analysis, which allowed the isolation of AA. The absorption spectrum and the isolated active compound retention time coincided with the AA I commercial standard, used in the chromatographic analysis. The main active compound isolated from *Aristolochia* genus plants is the AA that is structurally a mixture of nitro-phenanthrene and carboxylic acids, of which there are more than 50 analogous, among them the main constituents are: AAI, AA II, AA C, AA D, AA 7-OH and AA (Pacheco et al., 2012).

Thin layer chromatography methods HPLC-UV and HPLC-MS have been described for the analysis of AA, using only one or two substances as reference. Most reported analyses have been concentrated in AA analogues, mainly due the primary toxicity of the *Aristolochia* spp (Koh et al., 2006a, Koh et al., 2006b, Yuan et al., 2007a, Yuan et al., 2007b).

According to the chromatographic profiles results obtained by HPLC, AA was detected in the stem extract of *A. sprucei*, but not detected in the leaves extract. Nonetheless, variability in AA concentrations can exist, depending on different factors, such as: place, year season and part of the plant collected or sample analysis technique (Yuan et al., 2007a, Yuan et al., 2007b).

The AA structure isolated from *A. sprucei* was defined based in the ^1^H and ^13^C NMRs and through comparison with data described in the literature (Mors et al., 2000).

AA isolated from *A. sprucei* inhibited both *B. asper* and *B. jararacussu* venom myotoxic activities, and the respective venom isolated myotoxins MTX-II and BthTX-I. This work results partially agree with the ones from Vishwanath and Gowda (1987), who isolated AA from *Aristolochia* spp that interacted with the main PLA_2_s of *Vipera russelli* venom. The AA was a competitive inhibitor with a K_i_ of 9.9 × 10^−4^ M with phosphatidylcholine as substrate. The direct and indirect hemolysis inhibition was greater when compared to the enzymatic activity under phosphatidylcholine. It inhibited the edema forming activity produced by the PLA_2_s of *V. russelli*, but it did not inhibit other toxic activities produced by these PLA_2_s.

Some researchers, using amino acid chemical modifications, antibodies, and mutagenesis against specific domains of the svPLA_2_s, have shown the existence of functional separation between the enzymatic activity and the toxic activity of some svPLA_2_s, due to the presence of structural changes in domains responsible for these effects (Chioato and Ward, 2003, Lomonte et al., 1992, Rucavado and Lomonte, 1996, Soares et al., 2004, Soares and Giglio, 2003, Ward et al., 2002).

The AA, besides inhibiting the *B. asper* venom phospholipase activity, efficiently inhibited the myotoxic activity from *B. jararacussu, B. asper*, BthTX-I and MTX-II in a 1:30 ratio (w/w).

The absence of significant effects induced by AA over secondary structures of the myotoxins MjTX-I (Lys49-PLA_2_ homologue) and BthTX-II (Asp 49-PLA_2_) on CD analysis suggest that AA causes no significant structural changes in these proteins. In fact, previous crystallographic data showing that AA binds in a region exposed to the solvent for a Lys49-PLA_2_ structure with no relevant structural changes, is in agreement with the results found here (Fernandes et al., 2015a, Fernandes et al., 2015b).

In 1987, Vishwanath and coworkers have observed that AA binds to svPLA_2_s of *Vipera russelli*, even in the absence of substrate. However, the binding of AA to the free enzyme is reduced in comparison to the substrate-enzyme complex, which suggests the noncompetitive nature of the inhibition.

The most robust svPLA_2_s-like mechanism of action proposed to date (Dos Santos et al., 2009), describes that the myotoxicity effect triggered by Lys49-PLA_2_ may occurs as a result of dimerization (Dos Santos et al., 2011, Fernandes et al., 2014, Murakami and Arni, 2003), involving an allosteric transition and two different sites for interaction with the target membrane: i) the MDoS (Membrane-Docking Site) formed by the basic residues cluster (typically Lys20; Lys115 and Arg118 residues) and ii) the MDiS (Membrane-Disruption Site) formed by a hydrophobic cluster (typically Leu 121 and Phe 125 residues).

A partial neutralization of the myotoxic activity by AA and caffeic acid over PrTX-I from *B. pirajai*, a Lys49-PLA_2_ homologue, was observed. The PrTX-I/AA crystalized complex showed interactions in distinct sites, in which the AA was coupled on the dimer MDiS proximity (Fernandes et al., 2015a, Fernandes et al., 2015b).

The molecular docking study in this work presents AA most favorable conformations found on docking simulations performed with the following targets, the Lys49-PLA_2_s MTX-II, BthTX-I, MjTX-I and the Asp 49-PLA_2_ BthTX-II.

BthTX-II was the only Asp 49-PLA_2_ of this work, docking simulations showed AA interacting in the catalytic site of the toxin with a ΔG of −5.26 kcal/mol and hydrogen bond formation with His 48.

The best hits produced in the AA/MTX-II run exhibited an interaction between the NO_2_ group of the AA and the Lys-15 and Lys-115 in both dimer units, with hydrogen bonds formation and an average ΔG of −9 kcal/mol (Fig. 7). The interaction pattern observed in this case could explain the AA inhibition effect over the MTX-II in the myotoxicity assays, in what the MDoS impairment may be the cause of MTX-II myotoxic activity blockage by the AA.

Because MjTX-I presents an oligomeric conformation that is dependent on physicochemical conditions (Dos Santos et al., 2011), the monomeric conformation was opted to be used. Using this configuration, the best hits showed the ligand-protein interacting with residues of the MDoS, hydrophobic channel and C-terminus of the MjTX-I (Fig. 7). In the MDoS docked pose, the AA interacted with Lys115 and Lys 16, whereas in the hydrophobic channel, there was hydrogen bond formation between the AA and Lys49.

The docking simulations performed with BthTX-I in the dimeric form and AA, revealed an interaction with hydrogen bonds and a ΔG in the −9 kcal/mol range. As demonstrated here (Fig. 7), the best hits interacted directly with the MDoS, evidenced by the hydrogen bonds formed between the AA and the following residues in both chains: Lys115, Lys20, and Arg118. This stable interaction is possibly the reason for the protective effect of AA over the BthTX-I in the myotoxicity assays, which by blocking the MDoS may be preventing the BthTX-I interaction with membranes. Taking together, the docking experiments with different Lys49-PLA_2_ homologues and AA showed the potentiality of this ligand to interact with MDoS region, besides the MDiS region pointed by previous crystallographic data (Fernandes et al., 2015a, Fernandes et al., 2015b). The residues that form MDoS (Lys20, Lys115 and Arg118) are exposed to the solvent and its electrostatic and hydrophobic interactions with AA do not cause relevant structural changes, as observed in circular dichroism spectroscopy of MjTX-I with AA. These data are also in agreement with recent structural and functional studies that pointed out the essential role of the interaction between MDoS residues and different cinnamic acid derivatives (De Lima et al., 2017).

## 5 Conclusion

The previous reported antivenom activity of AA (Bhattacharjee et al., 2017, Carvalho et al., 2013, Ebrahimi et al., 2018, Kankara et al., 2020, Vishwanath et al., 1987), and the herein observed inhibitory actions of AA from *Aristolochia sprucei* over bothropic venoms and the isolated toxins, suggest that the AA may be a broad-spectrum inhibitor of snake venom toxins, highlighting this compound biotechnological potential. Further development using AA as a starting model in rational design approaches could generate optimized, more potent and safer derivatives, holding promises for future antivenom therapies. In addition, structural characterization by co-crystallization and kinetics functional studies of the AA/svPLA_2_s complexes analyzed here will be of critical importance to construct a better understanding about the mechanism of action of this inhibitor, as well as, useful in the of design new drugs leads with different action in different physiopathology processes.

## Ethical statement

All procedures involving animals were carried out in accordance with the Brazilian College of Animal Experimentation (COBEA) rules and with the approval of the Ethics Committee in the Use of Animals of the São Paulo University (CEUA No. January 08, 1096.53.1).

## CRediT authorship contribution statement

**Isela I. González Rodríguez:** Conceptualization, Methodology, Formal analysis. **Aleff F. Francisco:** Formal analysis, Writing - original draft. **Leandro S. Moreira-Dill:** Formal analysis. **Aristides Quintero:** Conceptualization, Methodology. **César L.S. Guimarães:** Formal analysis. **Carlos A.H. Fernandes:** Formal analysis, Writing - original draft. **Agnes A.S. Takeda:** Formal analysis. **Fernando B. Zanchi:** Formal analysis, Writing - original draft. **Cléopatra A.S. Caldeira:** Formal analysis. **Paulo S. Pereira:** Formal analysis, Writing - original draft. **Marcos R.M. Fontes:** Conceptualization, Methodology, Writing - original draft. **Juliana P. Zuliani:** Conceptualization, Methodology, Writing - original draft. **Andreimar M. Soares:** Conceptualization, Methodology, Writing - original draft.

## Declaration of competing interest

The authors declare that they have no knowledge competing financial interests or personal relationships that could have appeared to influence the work reported in this paper.
